# The Banana *MaWRKY18*, *MaWRKY45*, *MaWRKY60* and *MaWRKY70* Genes Encode Functional Transcription Factors and Display Differential Expression in Response to Defense Phytohormones

**DOI:** 10.3390/genes13101891

**Published:** 2022-10-18

**Authors:** Sergio Garcýa-Laynes, Virginia Aurora Herrera-Valencia, Lilia Guadalupe Tamayo-Torres, Verónica Limones-Briones, Felipe Alonso Barredo-Pool, Fray Martin Baas-Espinola, Angel Gabriel Alpuche-Solís, Carlos Puch-Hau, Santy Peraza-Echeverria

**Affiliations:** 1 Unidad de Biotecnología, Centro de Investigación Científica de Yucatán, Calle 43 No. 130 × 32 y 34, Colonia Chuburná de Hidalgo, Mérida 97205, Yucatán, Mexico; 2 División de Biología Molecular, Instituto Potosino de Investigación Científica y Tecnológica A. C., Camino a la Presa San José 2055, Col. Lomas 4 Sección, San Luis Potosí 78216, San Luis Potosí, Mexico; 3 Departamento de Recursos del Mar, Unidad Mérida, Centro de Investigación y de Estudios Avanzados del Instituto Politécnico Nacional (CINVESTAV-IPN), km. 6, Antigua Carretera a Progreso, Apdo. Postal 73-Cordemex, Mérida 97310, Yucatán, Mexico

**Keywords:** banana, transcription factor, WRKY, defense phytohormones, salicylic acid, methyl jasmonate, SAR, ISR, broad-spectrum resistance

## Abstract

WRKY transcription factors (TFs) play key roles in plant defense responses through phytohormone signaling pathways. However, their functions in tropical fruit crops, especially in banana, remain largely unknown. Several *WRKY* genes from the model plants rice (*OsWRKY45*) and *Arabidopsis* (*AtWRKY18*, *AtWRKY60*, *AtWRKY70*) have shown to be attractive TFs for engineering disease resistance. In this study, we isolated four banana cDNAs (*MaWRKY18*, *MaWRKY45*, *MaWRKY60*, and *MaWRKY70*) with homology to these rice and *Arabidopsis* *WRKY* genes. The *MaWRKY* cDNAs were isolated from the wild banana *Musa acuminata* ssp. *malaccensis*, which is resistant to several diseases of this crop and is a progenitor of most banana cultivars. The deduced amino acid sequences of the four *MaWRKY* cDNAs revealed the presence of the conserved WRKY domain of ~60 amino acids and a zinc-finger motif at the N-terminus. Based on the number of WRKY repeats and the structure of the zinc-finger motif, *MaWRKY18* and *MaWRKY60* belong to group II of WRKY TFs, while *MaWRKY45* and *MaWRKY70* are members of group III. Their corresponding proteins were located in the nuclei of onion epidermal cells and were shown to be functional TFs in yeast cells. Moreover, expression analyses revealed that the majority of these *MaWRKY* genes were upregulated by salicylic acid (SA) or methyl jasmonate (MeJA) phytohormones, although the expression levels were relatively higher with MeJA treatment. The fact that most of these banana *WRKY* genes were upregulated by SA or MeJA, which are involved in systemic acquired resistance (SAR) or induced systemic resistance (ISR), respectively, make them interesting candidates for bioengineering broad-spectrum resistance in this crop.

## 1. Introduction

Banana is a staple food for more than 400 million people in the tropics and a major source of foreign currency for the leading producers [1]. Banana ranks as the most prolifically produced fruit in the world, with 145 million tons per year, representing an estimated value of USD$52 billion [2]. Similar to other monocultures, banana cultivars are affected by a broad range of pathogens, including viruses, bacteria, fungi, and nematodes, which can cause partial or total loss of fruit production [3,4]. Fungal pathogens are the most destructive in the banana industry today, and the most notorious of them are the soil-borne *Fusarium oxysporum* f.sp. *cubense* (Foc), the causal agent of Panama disease, and the air-borne *Pseudocercospora fijiensis*, the causal agent of black Sigatoka disease. Foc tropical race 4 (TR4) threatens to destroy all banana plantations of susceptible cultivars in the tropics. Fungicides are largely ineffective against Foc. *P. fijiensis* is spread in almost all banana-growing regions of the world and requires an intensive regime of fungicide applications to control it, with up to 66 applications per year in some locations [3,4,5]. This conventional strategy to control *P. fijiensis* is one of the most expensive in agriculture, surpassing US$500 million per year [6] and putting the health of plantation workers and the environment at risk. Taking into account all these biotic factors, there is an urgent need to develop disease-resistant genotypes that preserve the current agronomic quality features of banana fruits without using pesticides. A better understanding of the genes involved in disease resistance in banana should provide valuable tools for the genetic improvement of this crop.

Plants have developed two effective spatial immune responses to recognize and repel pathogens. The first comprises mainly receptor-like proteins (RLPs) or receptor-like kinases (RLKs) located at the plasma membrane. RLPs and RLKs perceive extracellular immunogenic patterns (ExIPs) derived from the pathogen or damaged host cell, activating extracellularly triggered immunity (ExTI) that halts pathogen spread [7,8]. ExTI is usually associated with programmed cell death at the infection site, known as the hypersensitive response (HR). The second immune response takes place inside the cell and occurs when an intracellular receptor, mainly a nucleotide-binding site and leucine-rich repeat (NBS-LRR) protein, perceives intracellular immunogenic patterns (InIPs) derived from the pathogen or damaged host cell, activating intracellularly triggered immunity (InTI), which can also result in an HR to stop pathogen spread [7,8].

The HR generally activates a disease resistance mechanism known as systemic acquired resistance (SAR), which is dependent on the accumulation of the defense hormone salicylic acid (SA) and is effective against biotrophic or hemibiotrophic pathogens. The SAR response activates several types of pathogenesis-related (PR) proteins with antimicrobial activities, providing broad-spectrum resistance in the entire plant that can last several days [9,10]. The hormone jasmonic acid (JA) also plays a major role in regulating plant defense responses against necrotrophic pathogens [11]. Moreover, beneficial microorganisms can activate induced systemic resistance (ISR) through JA and its conjugates, such as methyl-JA (MeJa), leading to broad-spectrum resistance [12]. Recent studies have shown that SA also plays a role in ISR [13,14,15].

Several TF families have been implicated in the regulation network of SAR or ISR, such as AP2/ERF, bHLH, bZIP, MYB, NAC, and WRKY [16,17]. These TFs bind to promoter elements of defense genes, regulating their expression as either activators or repressors of transcription. WRKY TFs play a prominent role in the activation of immune responses. These TFs belong to a large plant gene family of transcriptional regulators, which are defined by the highly conserved WRKY domain (~60 amino acid residues in length) composed of a conserved WRKYGQK motif at the N-terminus and a zinc-finger motif at the C-terminus [18]. WRKY TFs recognize the W-box cis-acting element (TTGACY) in the promoters of their target genes. WRKY proteins are classified into three major groups based on the number of WRKY domains and the type of zinc finger motif [19]. Group I contains two WRKY domains in tandem and a C2H2 zinc finger type, whereas groups II and III each possess only one WRKY domain with a C2H2 zinc finger type and a C2HC zinc finger type, respectively. Group II WRKY proteins are further classified into five subgroups (IIa to IIe) by phylogenetic analysis [18]. Furthermore, several WRKY proteins are responsive to defense-related hormones, such as SA or JA, leading to disease resistance. Moreover, overexpression of *WRKY* genes can enhance disease resistance against a broad range of pathogens [18,20]; for example, the overexpression of rice *OsWRKY45* increased resistance against biotrophic and hemibiotrophic pathogens such as the bacterium *Xanthomonas oryzae* and the fungus *Magnaporthe grisea*, respectively, in transgenic rice [21]. Additionally, *Arabidopsis *AtWRKY70**, which is phylogenetically closely related to *OsWRKY45*, enhanced resistance to the bacterial pathogens *Erwinia carotovora* and *Pseudomonas syringae* and to the fungal pathogen *Erysiphe cichoracearum* in transgenic *Arabidopsis* [22,23]. Both genes belong to group III. Another example is *Arabidopsis *AtWRKY18**, whose overexpression increased resistance to *P. syringae* [24]. Moreover, *Arabidopsis* *AtWRKY60* enhanced the DNA-binding activity of *AtWRKY18* [25]. The *AtWRKY18* and *AtWRKY60* genes are phylogenetically closely related and belong to subgroup IIa of the WRKY family (Xu et al., 2006). Interestingly, homologs of *AtWRKY18* and *AtWRKY60* genes in banana were upregulated in response to the fungus *P. fijiensis* [26]. These reports and others make *WRKY* genes interesting candidates for the genetic improvement of crops.

In banana, genome-wide analyses have identified 164 members of the WRKY family, and transcriptomic analyses have revealed a possible involvement of members of groups II and III in disease resistance [26,27,28,29]. Although substantial advances have been made in the annotation of *WRKY* genes in banana and their expression analysis in response to biotic and abiotic stress, our understanding of their function as TFs and their regulation by defense hormones is still very limited. To expand the current knowledge of banana *WRKY* genes, in the present study, we isolated the cDNAs of four banana WRKY homologs, which we named *MaWRKY18*, *MaWRKY45*, *MaWRKY60*, and *MaWRKY70* due to their close phylogenetic relationships with their counterparts in *Arabidopsis* and rice. A comprehensive structural analysis of these banana *WRKY* genes was carried out, and their protein subcellular locations were determined. Furthermore, their function as TFs was determined, along with their expression profiles in response to the defense hormones SA and MeJA. The novel insights provided in this study represent a foundation for further functional analyses of these banana *WRKY* genes, which may lead to engineering disease resistance in this crop.

## 2. Materials and Methods

### 2.1. Plant Material and Phytohormone Treatments

Leaves of *Musa acuminata* ssp. *malaccensis* (ITC code: 1345) plants growing outdoors in leptosol soil at the Centro de Investigación Científica de Yucatán A. C. were used for RNA extraction and cloning of banana WRKY cDNAs. A floating leaf disc assay [30] was used for hormone treatments. Briefly, fully expanded young leaves next to the furled leaf of *M. acuminata* ssp. *malaccensis* adult plants were used to collect leaf fragments in the morning (9 a.m./26 °C), which were disinfected as described by Rodríguez-García et al. [31]. Leaf fragments were then cut into 10 circular pieces of 1.5 cm diameter and floated (adaxial surface facing up) on 30 mL of 5 mM SA [32] or 100 µM MeJA [33] in 10 cm diameter Petri dishes; both solutions contained 0.025% Silwet-L77 and 0.5% ethanol. For the control, the leaf discs were floated on 30 mL of 0.025% Silwet-L77 and 0.5% ethanol solution. All Petri dishes had 10 leaf discs, each excised from an independent plant. Three biological replicates were used for each hormone treatment, and three biological replicates were used for the control. Samples were incubated at 26 ± 1 °C in a growth room with a 16-h photoperiod (light intensity of 100 mmol m^–2^ s^–1^) for 6 h, and collected and stored at −80 °C until use for total RNA extraction.

### 2.2. BLAST Searches

The amino acid sequences of OsWRKY45 (GenBank accession number AK066255) [34] and *AtWRKY70* (GenBank accession number AF421157) [22] from rice and *Arabidopsis*, respectively, were used as queries to search (E-value cutoff of −1) the proteome of the wild species *M. acuminata* ssp. *malaccensis* [26] using the BLASTP algorithm at the Phytozome v12.1 database (https://phytozome.jgi.doe.gov/pz/portal.html, accessed on 20 January 2021). We also included the banana WRKY sequences GSMUA_Achr3T13440 and GSMUA_Achr7T05200 as queries in BLASTP searches, as they have been shown to be upregulated by the pathogen *P. fijiensis* and have been reported to be closely related to *AtWRKY18* and *AtWRKY60* [26]. In this case, the BLASTP searches were carried out at the Phytozome v12.1 database (accessed on 20 January 2021) using the *Arabidopsis* proteome. The four banana *WRKY* genes were named based on their *Arabidopsis* or rice *WRKY* gene counterparts, with the prefix ‘Ma’ indicating *M. acuminata*.

### 2.3. RT-PCR and Cloning of MaWRKY cDNAs

The Illustra Nucleon PhytopureTM kit (GE Healthcare Life Science, Chicago, IL, USA) was used to extract total RNA from leaf tissue of the wild banana *M. acuminata* ssp. *malaccensis* following the manufacturer’s instructions. Total RNA concentration and purity were determined using a NanoDrop Lite Spectrophotometer (Thermo Fisher Scientific, Waltham, MA, USA). Total RNA integrity was determined by agarose gel electrophoresis stained with ethidium bromide. The nucleic acid extract was treated with TURBO^TM^ DNase (Thermo Fisher Scientific, Waltham, MA, USA), and then first-strand cDNA synthesis was performed using 5 µg of total RNA employing SuperScript™ III (Thermo Fisher Scientific) according to the manufacturer’s protocol. Specific primers for the predicted coding sequences (CDSs) of the *MaWRKY18*, *MaWRKY45*, *MaWRKY60*, and *MaWRKY70* genes were designed (Table S1) and used for RT-PCR amplification using the Expand™ Long Template PCR System (Merck, Darmstadt, Germany). The cycling conditions were 95 °C for 3 min, followed by 35 cycles of 95 °C for 10 s, 59 °C for 30 s, and 72 °C for 1 min. The PCR products were cloned into the pGEM-T Easy vector (Promega, Madison, WI, USA) and sequenced on both strands by Macrogen, Inc. (https://dna.macrogen.Com, accessed on 8 February 2020). In addition, specific primers (Table S2) upstream of the stop codon of each CDS were designed for mapping the 3′ cDNA ends using the SMARTer^®^ RACE Kit (Takara Bio USA, San Jose, CA, USA) according to the manufacturer’s protocol. The PCR products were cloned and sequenced as described above. The sequences were edited using the BioEdit software v7.2. (Informer Technologies, Inc., Los Angeles, CA, USA) [35]. The cDNA sequences of *MaWRKY18*, *MaWRKY45*, *MaWRKY60*, and *MaWRKY70* were deposited in GenBank with the accession numbers OP186309, OP186310, OP186311, and OP186312, respectively.

### 2.4. Gene Structure, Identification of Conserved Protein Motifs, Protein Modeling, and Phylogenetic Tree Construction

The 3′ UTR (Untranslated Region), exon, and intron regions of banana *WRKY* genes were illustrated using the Exon-Intron Graphic Maker v4.0 platform (http://wormweb.org/exonintron, accessed on 8 June 2021). The *WRKY* CDS was translated with the Translate function of the BioEdit software v7.2., (Informer Technologies, Inc., Los Angeles, CA, USA) [35]. The identification of conserved motifs was performed using the ScanProsite Tool from the Expasy Bioinformatics Resource Portal (https://prosite.expasy.org/scanprosite/, accessed on 13 February 2021) with the default settings. The amino acid identity between sequences was determined with the Clustal Omega program (https://www.ebi.ac.uk/Tools/msa/clustalo/, accessed on 18 February 2021). The presence of cis-acting DNA regulatory elements in the putative promoter region (1500 bp upstream of the start codon) of each banana *WRKY* gene was detected using the databases PlantCARE [36] (http://bioinformatics.psb.ugent.be/webtools/plantcare/html/, accessed on 3 April 2021) and PlantPAN v3.0 [37] (http://plantpan.itps.ncku.edu.tw/, accessed on 5 April 2021) with the default settings. Homology modeling of protein structures was carried out using the SWISS-MODEL suite [38] (https://swissmodel.expasy.org/, accessed on 9 April 2021), and 3D models were edited with the PyMol program v2 (https://pymol.org/2/, accessed on 10 April 2021). Multiple sequence alignments were performed using the Clustal Omega program with the default parameter settings. Identical and similar amino acids were indicated with black and gray shading, respectively, using the Boxshade program (http://www.ch.embnet.org, accessed on 12 April 2021). A phylogenetic tree was constructed using the neighbor-joining (NJ) method with 1000 bootstrap replications, employing the Molecular Evolutionary Genetics Analysis (MEGA) v11.0.10 package [39] (http://www.megasoftware.net/, accessed on 13 April 2021). The phylogenetic tree was edited with the FigTree v1.4.4 program (http://tree.bio.ed.ac.uk/software/figtree, accessed on 14 April 2021). The banana *MaWRKY18*, *MaWRKY45*, *MaWRKY60*, and *MaWRKY70* protein sequences plus 70 members of the *Arabidopsis* WRKY family were used for phylogenetic tree construction.

### 2.5. Subcellular Localization Assay

The CDSs of the *MaWRY18*, *MaWRY45*, *MaWRY60*, and *MaWRKY70* cDNAs were fused in frame to the 3′ end of the GFP reporter gene present in the pAVA121 vector [40]. Onion epidermal cells were transiently transformed with the particle bombardment method [40] using a PDS-1000/He^TM^ system (Bio-Rad, Hercules, CA, USA). Green fluorescence was observed with an AxioScope A1 epifluorescence microscope (Carl Zeiss, Oberkochen, Germany), and image editing was performed with ZEN Edition Blue imaging software (Carl Zeiss, Oberkochen, Germany).

### 2.6. Yeast One-Hybrid Assay

The transcriptional activity of *MaWRKY18*, *MaWRKY45*, *MaWRKY60*, and *MaWRKY70* was determined by yeast one-hybrid assay using the pGBKT7 vector and the Y2HGold yeast strain (Takara Bio USA, San Jose, CA, USA). The pGBKT7 plasmid contains the GAL4 DNA-binding domain (BD) under the control of the ADH1 promoter and the TRP1 nutritional marker for selection in yeast, whereas the yeast Y2HGold strain contains the reporter genes AUR1-C, ADE2, and HIS3. The growing conditions used for the yeast Y2HGold strain are described in the Matchmaker^®^ Gold Yeast Two-Hybrid System User Manual (Takara Bio USA). The CDSs of *MaWRKY18*, *MaWRKY45*, *MaWRKY60*, and *MaWRKY70* cDNAs were PCR amplified using primers designed for In-Fusion^®^ cloning (Table S1) and cloned into the pGBKT7 vector by recombination using the In-Fusion^®^ HD Cloning Plus kit (Takara Bio USA) following the manufacturer’s instructions. The generated constructs were sequenced as described above and then used to transform the Y2HGold yeast strain, as described in the Matchmaker^®^ Gold Yeast Two-Hybrid System User Manual (Takara Bio USA). To test the transactivation activity, yeast strains harboring the recombinant and empty plasmids were streaked on synthetic dropout (SD) medium (-Trp, -His, -Ade) or YPDA medium supplemented with 200 ng/mL aureobasidin A. We used the papaya ERF transcription factor CpERF7 as a positive control [41].

### 2.7. RT-qPCR

Total RNA extraction and cDNA synthesis from floating leaf discs treated with SA or MeJA hormones were performed as previously described in Section 2.3. RT-qPCR was performed using Maxima SYBR Green/ROX qPCR master mix (Thermo Fisher Scientific) and specific primers for the *MaWRKY18*, *MaWRKY45*, *MaWRKY60*, and *MaWRKY70* genes (Table S1). The cycling conditions were 95 °C for 10 min, followed by 40 cycles of 95 °C for 10 s, 57 °C for 30 s and 72 °C for 30 s. Banana Ma25S gene expression was used as an internal standard [42] to normalize the expression of the banana WRKY genes, and the 2^−ΔΔCt^ method [43] was used to calculate the relative gene expression. In addition, specific primers for the banana MaDLO1 [32] and MaLOX1 [33] genes were used in the RT-qPCR assays as controls (Table S1). RT-qPCR was carried out using the StepOnePlus™ Real-Time PCR System (Thermo Fisher Scientific). Three biological replicates (each one with three technical replicates) and two experimental repetitions were performed for statistical analysis. The Anderson–Darling statistic was used to determine whether the data meet the assumption of normality for a *t*-test, and then a Student’s *t*-test was applied using the Minitab software v.17, (Informer Technologies, Inc., Los Angeles, CA, USA).

## 3. Results

### 3.1. Structural and Phylogenetic Analysis of the MaWRKY18, MaWRKY45, MaWRKY60, and MaWRKY70 Genes

BLASTP searches revealed that the best banana sequence hits to OsWRKY45 and AtWRKY70 were GSMUA_Achr5P07490 (E value = 1.78 × 10^−32^) and GSMUA_Achr1P27980 (E value = 2.98 × 10^−25^), respectively, whereas the best BLASTP hits for banana GSMUA_Achr3P13440 and GSMUA_Achr7P05200 in the *Arabidopsis* proteome were AtWRKY18 (E value = 1.81 × 10^−56^) and AtWRKY60 (E value = 1.76 × 10^−58^), respectively (Tables S3 and S4). The CDSs of the four banana WRKY cDNAs ranged from 828 (*MaWRKY18*) to 933 bp (*MaWRKY70*) in length (Table 1) (Figures S1–S4).

**Figure 1:**
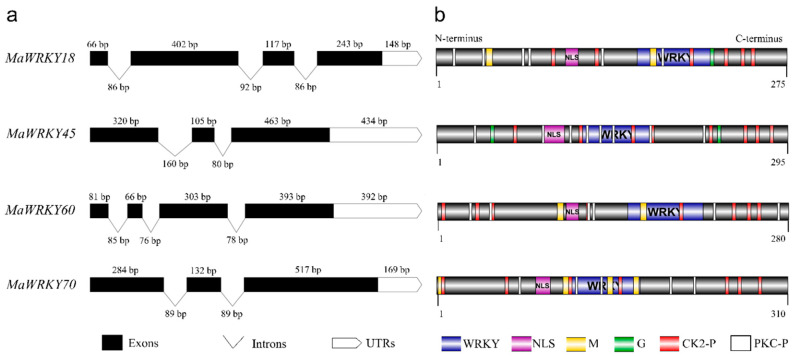
Schematic representation of *MaWRKY18*, *MaWRKY45*, *MaWRKY60*, and *MaWRKY70* cDNAs and their protein sequence structures. (**a**) Exon/intron and 3′ UTR composition. (**b**) Protein domains and posttranslational modification predictions. UTRs, untranslated regions; NLS, nuclear localization signal; WRKY, WRKY domain; M, myristoylation; G, glycosylation; CK2-P, CK2-phosphosites; PKC-P, PKC-phosphosite.

**Figure 2:**
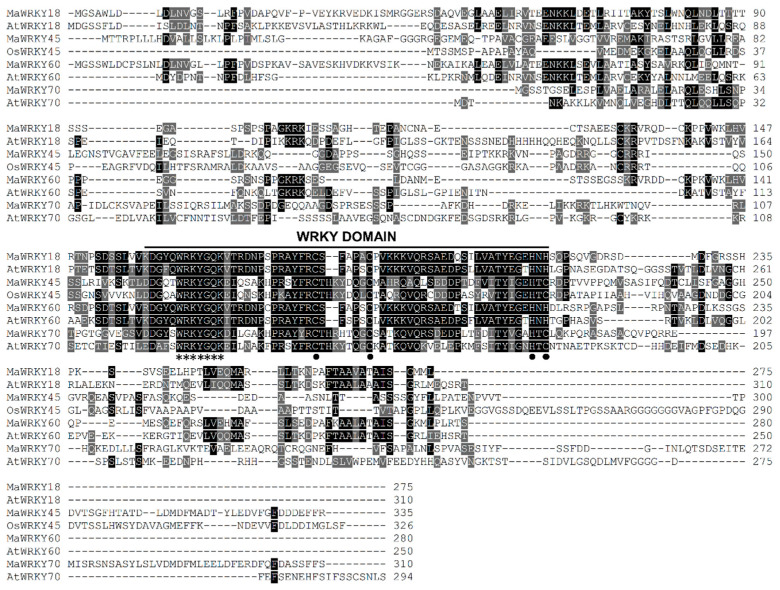
Multiple sequence alignment of the proteins MaWRKY18, MaWRKY45, MaWRKY60, MaWRKY70, and their homologs from rice and *Arabidopsis*. Identical and similar amino acids are shaded in black and gray, respectively. The amino acids of the WRKYGQK and zinc-finger motifs are highlighted with asterisks and black circles, respectively.

*MaWRKY18* and *MaWRKY60* contained the highest numbers of exons and introns, with 4 and 3, respectively, while *MaWRKY45* and *MaWRKY70* contained the lowest numbers of exons and introns, with 3 and 2, respectively (Figure 1a). The 3′ UTR lengths varied from 148 (*MaWRKY18*) to 434 (*MaWRKY45*) bp. The amino acid sequence lengths ranged from 275 (*MaWRKY18*) to 310 (*MaWRKY70*) (Table 1), while the protein sequence similarities among them ranged from 16% (*MaWRKY60* vs. *MaWRKY70*) to 67.9% (*MaWRKY18* vs. *MaWRKY60*) (Table S5). The deduced amino acid sequences of the four *MaWRKY* cDNAs revealed the presence of the conserved WRKY domain of ~60 amino acids (Figure 1b and Figure 2), which comprises the canonical WRKYGQK and zinc-finger motifs. Based on the number of WRKY repeats and the structure of the zinc-finger motif [18], *MaWRKY18* and *MaWRKY60* were determined to belong to group II, while *MaWRKY45* and *MaWRKY70* were classified as members of group III. Moreover, the four banana WRKY proteins had a potential nuclear localization signal (NLS) at the N-terminus near the WRKY domain (Figure 1b). We also found potential phosphorylation sites in the four protein sequences (Figure 1b). All proteins except MaWRKY45 had posttranslational sites (PTSs) for myristoylation (Figure 1b). Glycosylation sites were also found for MaWRKY18 and MaWRKY45 proteins (Figure 1b).

**Figure 3:**
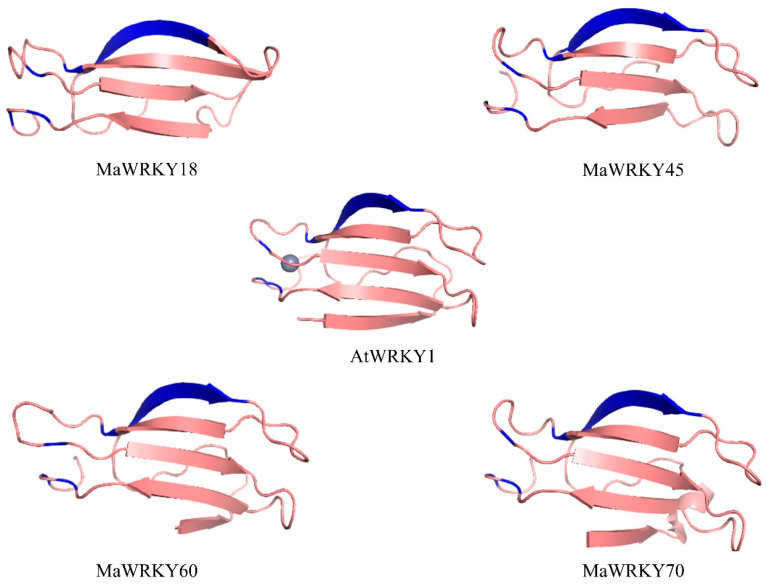
3D ribbon cartoon view of the WRKY domain of MaWRKY18, MaWRKY45, MaWRKY60, and MaWRKY70. The WRKYGQK β-sheets and the zinc-finger motifs are shown in blue. The zinc ion is presented for the *Arabidopsis* AtWRKY1 3D model (PDB accession code 2AYD).

**Figure 4:**
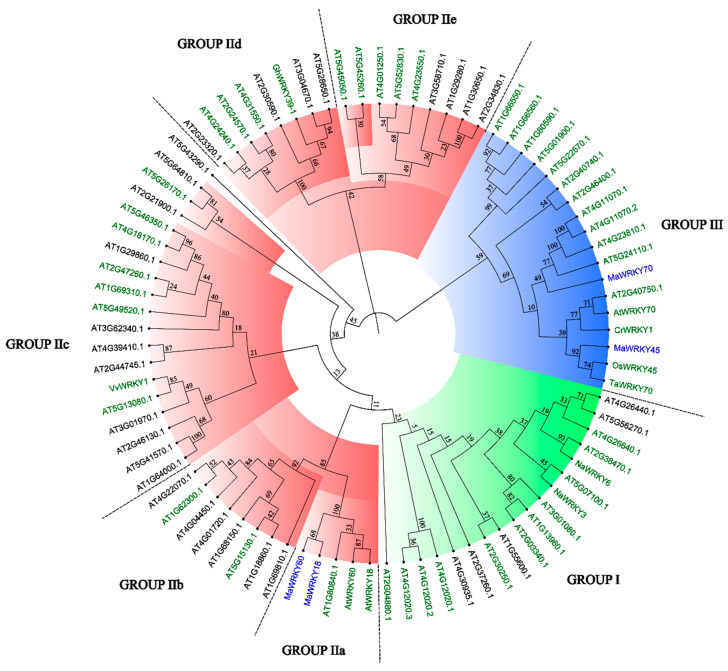
Neighbor-joining phylogenetic tree of MaWRKY18, MaWRKY45, MaWRKY60, and MaWRKY70 TFs, including the complete set of *Arabidopsis* WRKY TFs and other plant WRKY TFs with known functions. Groups I, II, and III of the WRKY TF family are highlighted in green, red, and blue, respectively. WRKY TFs with green names are involved in biotic stress responses. MaWRKY18, MaWRKY45, MaWRKY60, and MaWRKY70 are highlighted in blue. Numbers above branches are bootstrap values supporting the nodes based on 1000 bootstrap replications.

The 3D models of MaWRKY18, MaWRKY45, MaWRKY60, and MaWRKY70 (Figure 3) showed four antiparallel β-sheets comprising the WRKY DNA-binding domain similar to the WRKY domain of the *Arabidopsis* AtWRKY1 protein [44]. The phylogenetic tree (Figure 4) revealed that MaWRKY18 and MaWRKY60 clustered in clade IIa along with AtWRKY18 and AtWRKY60, while MaWRKY45 and MaWRKY70 clustered in clade III, with their homologs OsWRKY45 and AtWRKY70.

**Figure 5:**
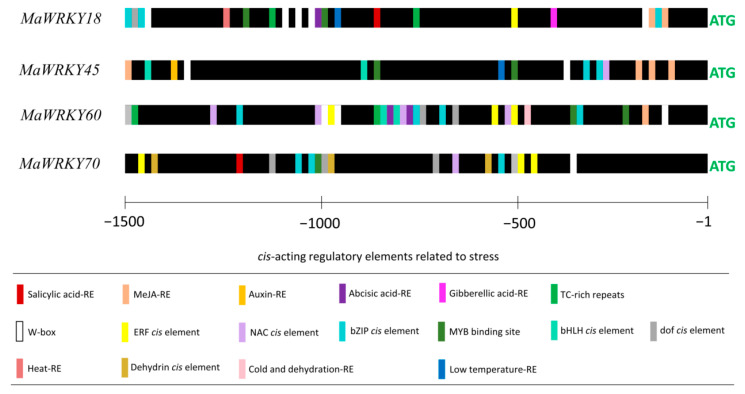
Predicted cis-acting regulatory elements in the promoter regions of *MaWRKY18*, *MaWRKY45*, *MaWRKY60*, and *MaWRKY70*. A region of 1500 bp upstream of the ATG start codon of each gene was used for the prediction of cis-acting regulatory elements. Bars with different colors represent the predicted cis-acting regulatory elements.

The promoter regions (1500 bp upstream of the start codon) of *MaWRKY18*, *MaWRKY45*, *MaWRKY60*, and *MaWRKY70* contained potential cis-acting regulatory elements (Figure 5). The four promoters contained potential hormone-responsive elements, such as SA [CCATCTTTTT], MeJA [TGACG], auxin [AACGAC], abscisic acid [CACGTG], and gibberellic acid [CCTTTTG]. The MeJA-responsive element was found in the promoter regions of *MaWRKY18*, *MaWRKY45*, and *MaWRKY60*. *MaWRKY45* had the highest number of MeJA-responsive elements, with four. Single SA-responsive elements were found in the promoter regions of *MaWRKY18* and *MaWRKY70*. All four gene promoters also had W-boxes [TTGACC], which are the sequences recognized by WRKY TFs. *MaWRKY18* and *MaWRKY60* had the highest numbers of W-boxes, with five and three, respectively, while *MaWRKY45* had two and *MaWRKY70* had only one. Other TF-responsive elements, such as NAC [CATGTG], ERF [TAAGAGCCGCC], MYB [CGGTCA], bZIP [TACGTA and CACGTG], dof [T/AAAAG], and bHLH [CANNTG], were also present in the four promoters (Figure 5). Moreover, cis-acting regulatory elements involved in heat [AGAAAATTCG], cold [CCGAAA], or dehydration [CAACTG] responses were found in the promoter regions of the four banana *WRKY* genes, suggesting their roles in the cross-talk between abiotic and biotic stress responses.

### 3.2. Subcellular Localization of MaWRK18, MaWRKY45, MaWRKY60, and MaWRKY70

**Figure 6:**
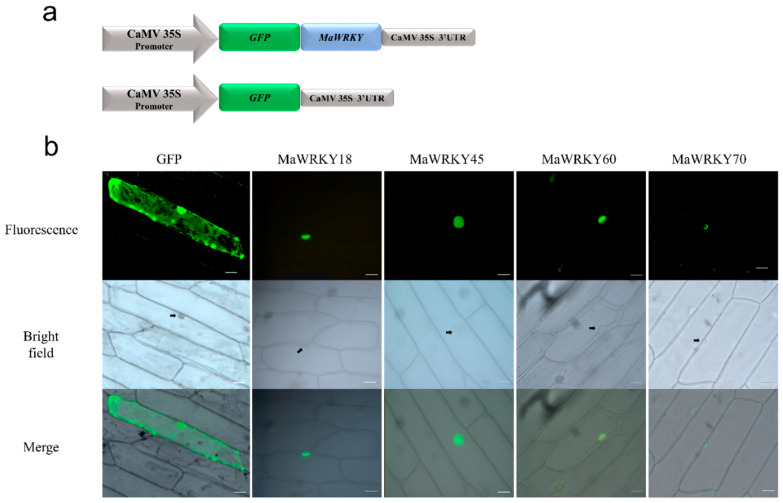
Subcellular localization of MaWRKY18, MaWRKY45, MaWRKY60, and MaWRKY70 proteins in onion epidermal cells. (**a**) Schematic representation of the expression cassettes used for the transient expression analyses. Gene expression is regulated by the CaMV 35S promoter and terminator present in the pAVA121 vector [40]. (**b**) The fluorescence of GFP fused with the MaWRKY proteins is localized in the nucleus, whereas the fluorescence of GFP alone used as a control is present in both the cytoplasm and nucleus. Scale bars: 5 μm.

As previously described, the four banana WRKY proteins were predicted to be located within the nucleus. To confirm whether these proteins were indeed localized in the nucleus of the cell, their coding sequences were fused in-frame to the GFP gene and transiently expressed in onion epidermal cells. We confirmed that these four MaWRKY proteins were localized inside the nucleus (Figure 6), indicating that these four MaWRKY proteins have roles in this cellular compartment.

### 3.3. Transactivation Activity of MaWRK18, MaWRKY45, MaWRKY60, and MaWRKY70 Transcription Factors

**Figure 7:**
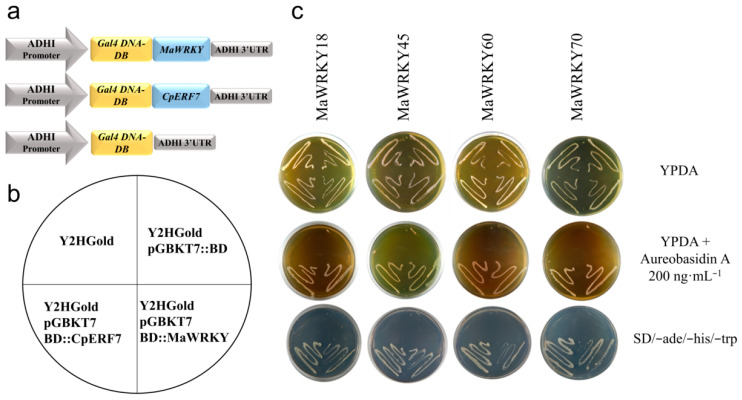
Transactivation activity analysis of MaWRKY18, MaWRKY45, MaWRKY60, and MaWRKY70 in Y2HGold yeast. (**a**) Schematic representation of the expression cassettes used for the transient expression analyses. Gene expression is regulated by the *ADH1* promoter and terminator present in the pGBKT7 vector. (**b**) The diagram shows the order in which the yeast strains were plated. (**c**) Yeast strains transformed with *MaWRKYs* fused with *Gal4* DNA-BD were grown in YPDA or YPDA supplemented with aureobasidin A or SD minimal medium (-Trp, -His, -Ade). The *Gal4* DNA BD::*CpERF7* construct [41] was used as a positive control in this assay.

We carried out a yeast one-hybrid assay to test the in vivo transactivation activity of the *MaWRKY18*, *MaWRKY45*, *MaWRKY60* and *MaWRKY70* proteins. The cDNA coding sequences of these four *MaWRKYs* were fused in-frame to the GAL4 DNA-binding domain (Figure 7a), resulting in fusion proteins capable of activating the transcription of three independent reporter genes (*AUR1-C*, *ADE2*, and *HIS3*) and promoting the growth of the Y2HGold yeast strain in either SD minimal medium (-Trp, -His, -Ade) or YPDA medium supplemented with the highly toxic drug aureobasidin A (Figure 7b,c). The two negative controls used in the yeast one-hybrid assay did not grow on YPDA medium supplemented with aureobasidin A or SD (-Trp, -His, -Ade) minimal medium, whereas the positive control CpERF7 did grow (Figure 7c). These results indicated that these MaWRKY proteins were functional TFs capable of positively regulating the transcription process in yeast.

### 3.4. Expression Profiles of the MaWRK18, MaWRKY45, MaWRKY60, and MaWRKY70 Genes in Response to Phytohormone Treatments

**Figure 8:**
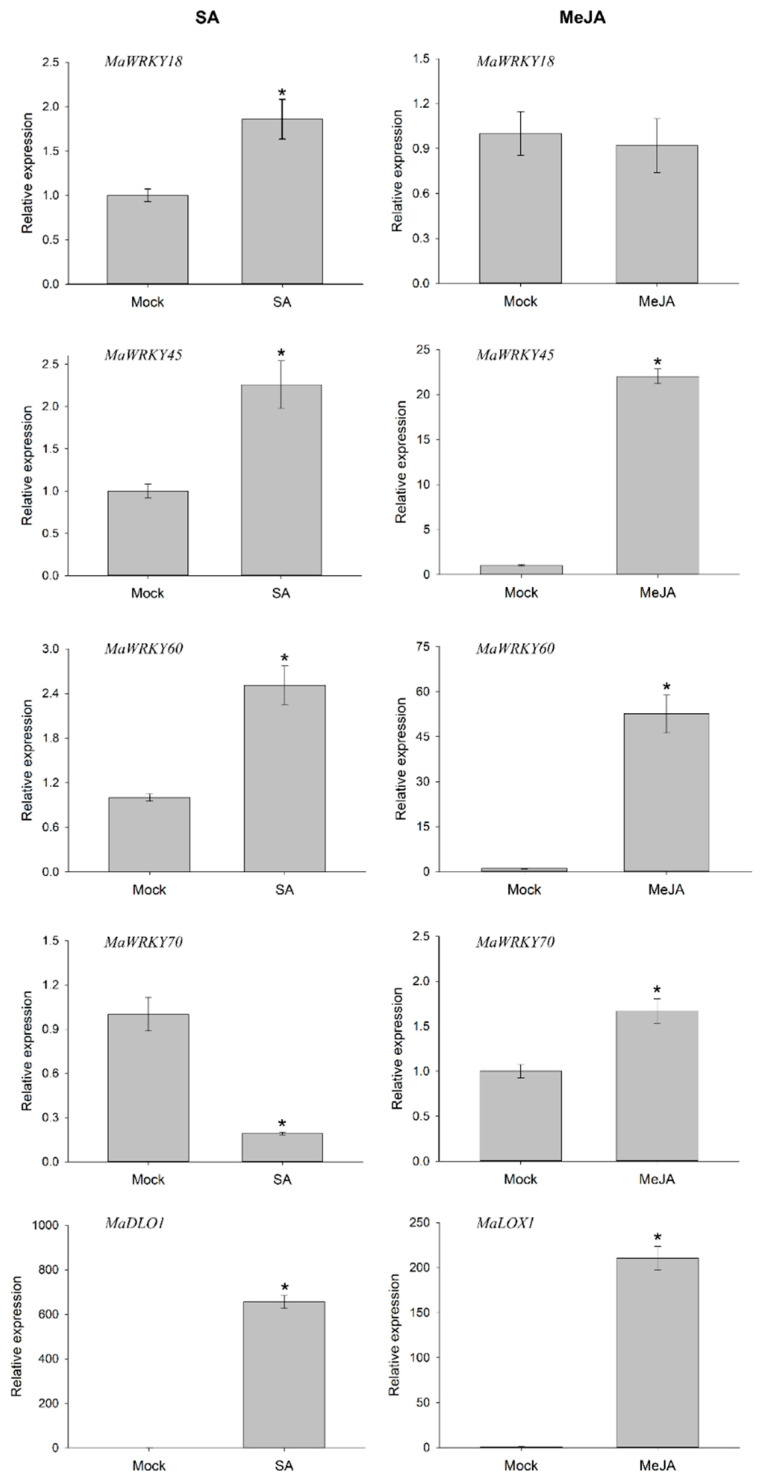
Transcription profiles of *MaWRKY18*, *MaWRKY45*, *MaWRKY60*, and *MaWRKY70* genes in response to SA and MeJA phytohormones. A floating disc assay was performed in either SA (5 mM) or MeJA (100 µM) phytohormones. The leaf discs were incubated for 6 h and collected for total RNA extraction. Gene expression was evaluated by quantitative real-time PCR. The banana *25S* gene was used as a reference gene for RT-qPCR analysis. The *MaDLO1* and *MaLOX1* genes were used as SA- and MeJA-responsive marker genes, respectively. The relative expression value was calculated by the 2^−ΔΔCT^ method [43]. The mean ± S.D. of three biological replicates is presented. Asterisks (*) indicate *p* ≤ 0.05 (*t* test).

To determine whether the *MaWRK18, *MaWRKY45*, *MaWRKY60**, and *MaWRKY70* genes were responsive to phytohormones involved in plant defense-signaling pathways, we performed an RT-qPCR analysis to measure their transcript levels when exposed to exogenous SA or MeJA after 6 h. The four genes were responsive to SA (Figure 8). The expression levels of *MaWRKY45* and *MaWRKY60* increased above 2-fold, while *MaWRKY18* expression increased 1.7-fold. In the case of *MaWRKY70*, its expression decreased by 5-fold. The expression of the SA-responsive marker *MaDLO1* increased significantly by 655-fold. The transcript levels in MeJA-treated samples exhibited higher expression levels than in samples treated with SA. The transcription levels of *MaWRKY45*, *MaWRKY60*, and *MaWRKY70* increased significantly, with *MaWRKY60* being the most responsive gene to MeJA treatment, with a 52-fold increase, whereas the *MaWRKY18* gene was not responsive to MeJA. As an MeJA-responsive marker, the *MaLOX1* gene was analyzed and showed a marked increase of 210-fold. These results suggest that the *MaWRKY18*, *MaWRKY45*, *MaWRKY60*, and *MaWRKY70* genes may play roles in SA or MeJA defense-signaling pathways.

## 4. Discussion

The *WRKY* genes are widespread in plant genomes, where they play a fundamental role in the transcriptional regulation of genes involved in growth, development, and stress responses [45,46]. Annotation of *WRKY* genes in angiosperm plants has shown that they belong to a large gene family ranging from 38 to 287 members in *Genlisea aurea* and *Glycine max*, respectively [47]. Despite these advances in *WRKY* gene annotations, only a small percentage of them have been functionally characterized, mainly in the model plants *Arabidopsis* and rice. In the case of economically important fruit crops, such as banana, with 164 *WRKY* genes annotated [29], only a few *WRKY* genes have been validated as TFs [48,49,50,51]. Cloning and elucidating the functional aspects of banana *WRKY* genes will expand our understanding of the multiple roles of these transcription factors and provide novel tools for genetic improvement.

OsWRKY45, AtWRKY18, AtWRKY60, and AtWRKY70 play pivotal roles in the resistance to fungal and bacterial pathogens [22,25,34]. Given the importance of these TFs in plant defense responses, in this study, we searched the banana genome for potential orthologs of these genes. The MaWRKY45 and MaWRKY70 proteins resulted in the best BLASTP hits using their counterparts from rice and *Arabidopsis* as queries, respectively. It has previously been shown that OsWRKY45 and AtWRKY70 are phylogenetically closely related [52]; interestingly, MaWRKY45 and MaWRKY70 also clustered in the same phylogenetic clade as OsWRKY45 and AtWRKY70, suggesting an evolutionary relationship and function in stress responses. In the case of MaWRKY18 and MaWRKY60, their best BLASTP hits were with AtWRKY18 and AtWRKY60 from *Arabidopsis*, respectively. They shared the features of the group IIa WRKY TFs and clustered with AtWRKY18 and AtWRKY60, which are phylogenetically closely related [25], implying a possible orthology for MaWRKY18 and MaWRKY60.

We identified multiple potential phosphorylation sites in the protein sequences of MaWRKY18, MaWRKY45, MaWRKY60, and MaWRKY70, even at the core of WRKY domains, which indicates that the activity of these banana WRKYs might be modulated via phosphorylation. Numerous WRKY TFs have been shown to be phosphorylated by mitogen-activated protein kinases, leading to enhanced binding to W-box elements and transcriptional activity [18]. We also identified N-myristoylation sites for these banana WRKY proteins. The number of characterized myristoylated proteins in plants is still limited [53], and to our knowledge, no myristoylated WRKY TF has been experimentally validated. Analysis of the myristoylome in *Arabidopsis* revealed that numerous membrane proteins involved in stress responses are myristoylated, suggesting that the myristoylome might function as a dynamic proteome in response to abiotic and biotic stresses [53]. Moreover, myristoylation of the BASP1 protein is required for its function as a transcriptional repressor by binding to the Wilms’ tumor 1 (WT1) transcription factor and other corepressor proteins [54]. By analogy, some myristoylated MaWRKY TFs could work in a similar manner. We also found NLS sites in all four banana WRKY proteins considered in this study and confirmed that these proteins were indeed localized in the nuclei of onion epidermal cells. These results are consistent with the nuclear localization of other plant WRKY TFs, including the banana WRKYs reported thus far [48,49,50,51,55]. Furthermore, we demonstrated that these four banana WRKYs were functional transcription factors in yeast since all of them were capable of activating RNA polymerase II for the transcription of three marker genes. Less than 5% of the WRKY genes found in the banana genome have been experimentally validated as TFs, and most of them are from the cultivar Cavendish [48,49,50,51]; this is the first report that assessed the transactivation activity of WRKY proteins from a wild banana (*M. acuminata* ssp. *malaccensis*), which is resistant to the most destructive diseases of this crop and is a progenitor of most banana cultivars [26].

Numerous putative cis-acting regulatory elements related to defense phytohormones were found in the promoter regions of all four banana *WRKY* genes, including SA and MeJA, which are two key phytohormones involved in SAR and ISR, respectively [56]. *MaWRKY18*, *MaWRKY45*, and *MaWRKY60* were upregulated in response to SA. This is in agreement with the expression profiles of *AtWRKY18*, *OsWRKY45*, and *AtWRKY60,* which were also upregulated by SA [24,34,57]. However, *MaWRKY70* was downregulated by SA, in contrast to *AtWRKY70,* which has been shown to be upregulated by this phytohormone [22]. Regarding MeJa, *MaWRKY45*, *MaWRKY60*, and *MaWRKY70* were upregulated in response to this phytohormone, unlike *MaWRKY18*, which was not responsive to MeJA. Interestingly, both *MaWRKY18* and *MaWRKY60* were upregulated by the fungal pathogen *P. fijiensis* in susceptible banana accessions [26]. Taking into consideration that *MaWRKY18* was upregulated by SA and *MaWRKY60* by both SA and MeJa, it is plausible that both phytohormones play roles in the banana–*P. fijiensis* pathosystem. In the case of *Arabidopsis AtWRKY18* and *AtWRKY60*, it remains to be seen whether these TFs respond to MeJA. Previous reports have shown that *OsWRKY45* and *AtWRKY70* were upregulated by MeJa [22,57]. These two TFs function as a convergent point of jasmonate- and salicylate-mediated signals in plant defense [22,48]. Moreover, *AtWRKY70* is regulated downstream of the master regulator of SAR, the transcription cofactor AtNPR1, while OsWRKY45 works independently of OsNPR1 [48], indicating that although these two TFs are phylogenetically closely related and function downstream of SA, they respond differently to NPR1. Thus, a similar scenario may occur in *MaWRKY45* and *MaWRKY70*. Moreover, the promoter regions of *MaWRKY18*, *MaWRKY45*, *MaWRKY60*, and *MaWRKY70* contained W-boxes, suggesting that the expression of these banana *WRKY* genes could be either autoregulated or cross-regulated by other MaWRKYs, as previously reported [58]. W-boxes are ubiquitous in the promoter regions of stress-inducible genes [59,60].

The fact that these four *MaWRKY* genes were responsive to phytohormones involved in SAR and ISR makes them interesting candidates for engineering broad-spectrum resistance in banana. Since constitutive overexpression of *WRKYs* can lead to adverse effects on agronomic traits [21,24,34], it is necessary to fine-tune their expression through a moderate constitutive or pathogen-inducible promoter to harness the full potential of *WRKY* genes for boosting the plant immune system without detrimental effects on growth and development. Promising advances in this direction have been made in rice with a moderate constitutive and pathogen-inducible promoter driving the expression of *OsWRKY45* [61,62]. Therefore, the use of these types of promoters in banana for driving the expression of *MaWRKY18*, *MaWRKY45*, *MaWRKY60*, and *MaWRKY70* may lead to triggering SAR or ISR, providing broad-spectrum resistance without affecting agronomic traits.

## 5. Conclusions

We isolated the cDNAs of four *WRKY* genes from a wild banana (*M. acuminata* ssp. *malaccensis*) and found that they belong to group II (*MaWRKY18* and *MaWRKY60*) and group III (*MaWRKY45* and *MaWRKY70*) of plant *WRKY* genes. Their proteins were located in the nuclei of onion epidermal cells and were functional TFs in yeast cells. Furthermore, these *MaWRKY* genes were responsive to the phytohormone SA, implying a possible role in SAR, while three of them were responsive to the phytohormone MeJA, suggesting a possible role in ISR. These results make *MaWRKY18*, *MaWRKY45*, *MaWRKY60*, and *MaWRKY70* interesting candidates for bioengineering broad-spectrum resistance in banana, using moderate constitutive or pathogen-inducible promoters to fine-tune their expression.

## Figures and Tables

**Table 1:** Relevant features of *MaWRKY18*, *MaWRKY45*, *MaWRKY60*, and *MaWRKY70* cDNAs and their deduced proteins.

Gene Name	Genbank Accession Number of cDNAs	CDS Length (bp)	3′UTR Length (bp)	Number of Exon/Intron	Chromosome Location No.	Protein Length (aa)	Domains	kDa	pI	Subcellular Location
*MaWRKY18*	OP186309	828	148	4/3	3	275	WRKY	30.31	6.61	Nucleus
*MaWRKY45*	OP186310	888	434	3/2	5	295	WRKY	32.36	5.34	Nucleus
*MaWRKY60*	OP186311	843	392	4/3	7	280	WRKY	30.66	8.80	Nucleus
*MaWRKY70*	OP186312	933	169	3/2	1	310	WRKY	34.52	5.54	Nucleus

## Data Availability

Not applicable.

## Supplementary Materials

The following supporting information can be downloaded at: https://www.mdpi.com/article/10.3390/genes13101891/s1, Table S1: List of primers for RT-PCR and RT-qPCR; Table S2: List of forward primers for 3′ UTR amplification using RACE; Table S3: Best BLASTP hits of rice OsWRKY45 and *Arabidopsis* AtWRKY70 protein sequences used as queries in the proteome of *M. acuminata* ssp. *Malaccensis*; Table S4: Best BLASTP hits of banana GSMUA_Achr3P13440 and GSMUA_Achr7P05200 protein sequences used as queries in the *Arabidopsis* proteome; Table S5: Amino acid sequence similarity (%) between banana MaWRKY18, MaWRKY45, MaWRKY60, MaWRKY70 and their respective homologs in *Arabidopsis* and rice; Figure S1: cDNA sequence of *MaWRKY18* and its deduced amino acid sequence; Figure S2: cDNA sequence of *MaWRKY45* and its deduced amino acid sequence; Figure S3: cDNA sequence of *MaWRKY60* and its deduced amino acid sequence; Figure S4: cDNA sequence of *MaWRKY70* and its deduced amino acid sequence.
