# Supplementary material for: The Banana MaWRKY18, MaWRKY45, MaWRKY60 and MaWRKY70 Genes Encode Functional Transcription Factors and Display Differential Expression in Response to Defense Phytohormones
Source: Genes (Basel). 2022 Oct 18;13(10):1891. doi: 10.3390/genes13101891 (PMC9602068; doi:10.3390/genes13101891)
Supplement: Supplementary file 1 [file genes-13-01891-s001.zip › genes-1943520-supplementary.pdf]

```
1     ATG GGA TCG GCT TGG TTG GAT CTC GAC CTC AAC GTT GGC TCG CTC    45
1      M   G   S   A   W   L   D   L   D   L   N   V   G   S   L     15

46    CGA TTC CCT GTC GAT GCT CCG CAA GTG TTC CCC GTG GAA TCC AAG    90
16     R   F   P   V   D   A   P   Q   V   F   P   V   E   S   K     30

91    CTT GTC GAG GAT AAG ATC TCG ATG AGA GGA GGC GAA AGA AGT GAT    135
31     L   V   E   D   K   I   S   M   R   G   G   E   R   S   D     45

136   GCT CAA GTT GAA GGT CTC GAG GCG GAG CTG ATT CGG GTG ACG GAG    180
46     A   Q   V   E   G   L   E   A   E   L   I   R   V   T   E     60

181   GAG AAC AAG AAA CTC GAT GAA ACG CTG CGA ATC ATC ACG GCG AAG    225
61     E   N   K   K   L   D   E   T   L   R   I   I   T   A   K     75

226   TAC ACC TCG CTT TGG AAC CAG CTG AAT GAT CTG ACG ACC ACC ACC    270
76     Y   T   S   L   W   N   Q   L   N   D   L   T   T   T   T     90

271   TCC TCT TCC GAG GGG GCG TCT CCC TCT CCC ACA CCT GCA GGG AAG    315
91     S   S   S   E   G   A   S   P   S   P   T   P   A   G   K     105

316   AGG AAG ATC GAG AGC TCC GCA GGC CAC ACC GAG CCG GCT AAC TGC    360
106    R   K   I   E   S   S   A   G   H   T   E   P   A   N   C     120

361   AAC GCG GAG TGC ACG TCG GCC GAG GAG TCC TGC AAG CGA GTC AGA    405
121    N   A   E   C   T   S   A   E   E   S   C   K   R   V   R     135

406   CAG GAT TGC AAA CCC CCG GTC TGG AAG CTT CAC GTT CGC ACC AAT    450
136    Q   D   C   K   P   P   V   W   K   L   H   V   R   T   N     150

451   CCA TCC GAT TCG AGT CTG GTC GTG AAA GAT GGG TAT CAA TGG AGG    495
151    P   S   D   S   S   L   V   V   K   D   G   Y   Q   W   R     165

496   AAG TAT GGT CAG AAG GTG ACA AGA GAC AAC CCC TCT CCA AGA GCT    540
166    K   Y   G   Q   K   V   T   R   D   N   P   S   P   R   A     180

541   TAC TTC CGG TGC TCC TTC GCG CCT GCC TGC CCG GTG AAG AAG AAG    585
181    Y   F   R   C   S   F   A   P   A   C   P   V   K   K   K     195

586   GTG CAA AGA AGC GCG GAG GAT CAA TCC ATC TTG GTG GCG ACG TAC    630
196    V   Q   R   S   A   E   D   Q   S   I   L   V   A   T   Y     210

631   GAA GGC GAG CAC AAT CAC AGC CAG CCT TCT CAG GTC GGA GAT CGC    675
211    E   G   E   H   N   H   S   Q   P   S   Q   V   G   D   R     225

676   AGT GAC ATG GAT TTC GGC AGG TCT TCG CAT CCC AAG TCA TCA GTG    720
226    S   D   M   D   F   G   R   S   S   H   P   K   S   S   V     240

721   TCG GAG GAA CTC CAT CCA ACT TTG GTG GAG CAG ATG GCT CGG TTG    765
241    S   E   E   L   H   P   T   L   V   E   Q   M   A   R   L     255

766   TTG ACC AAA AAT CCA GCC TTC ACA GCT GCT GTA GCT ACT GCC ATT    810
256    L   T   K   N   P   A   F   T   A   A   V   A   T   A   I     270

811   TCC GGG ATG ATG CTT TAA cca aaa atc cag cct tca cag ctg ctg    855
271    S   G   M   M   L   *                                         276

856   tag cta ctg cca ttt ccg gga tga tgc ttt aac caa aat cca ccc    900

901   cca aaa gat gcc atg aat tca agg ctt ctt ctc tgt gac gct ttc    945

946   tta tag gta aag gat aaa tga ttt gag ctc a                      976
```

Figure S1. cDNA sequence of *MaWRKY18* and its deduced amino acid sequence. The WRKY domain is highlighted in gray, while the WRKYGQK motif is highlighted in blue. The amino acids of the zinc-finger motif are highlighted in red. Putative NLS residues are highlighted in orange. The 148 bp of the 3’ UTR is in lower case letters. GenBank accession number: OP186309.

1	ATG	GAA	CAC	ACG	CCT	GTT	GTG	GCT	TGT	GGG	GAA	GCC	GAG	GAG	TCC	45
1	M	E	H	T	P	V	V	A	C	G	E	A	E	E	S	15
46	CTG	GTC	GGC	GGC	ACG	GTG	GTG	CGG	GAG	ATG	GCG	AAG	ATC	CGG	GCA	90
16	L	V	G	G	T	V	V	R	E	M	A	K	I	R	A	30
91	TCG	ACC	TCC	CGG	CTC	GGA	GTG	TTG	CTC	CGG	GAG	GCG	TTG	GAG	GGG	135
31	S	T	S	R	L	G	V	L	L	R	E	A	L	E	G	45
136	AAT	TCC	ACG	GTG	GGA	GCC	GTC	TTT	GAG	GAG	CTC	GAA	GGC	TCG	ATC	180
46	N	S	T	V	G	A	V	F	E	E	L	E	G	S	I	60
181	TCG	CGA	GCC	TTC	TCT	CTG	CTG	GAC	CGC	AAG	CAG	CAG	GGC	GGA	GAT	225
61	S	R	A	F	S	L	L	D	R	K	Q	Q	G	G	D	75
226	GGC	CCC	CCT	TCT	TCT	GAG	CAC	CGA	AGT	AGC	GAG	ATC	CCG	ACC	AAG	270
76	G	P	P	S	S	E	H	R	S	S	E	I	P	T	K	90
271	AAA	CGG	AAG	GTG	AAT	CCG	GCG	TGT	GAT	CGG	CGT	GGA	GGT	TGC	CGG	315
91	**K**	**R**	**K**	**V**	**N**	**P**	**A**	**C**	**D**	**R**	**R**	**G**	**G**	**C**	**R**	105
316	AGA	AGA	ATA	CAA	TCT	TCT	TCC	CTG	CGG	ATC	GTC	AAA	TCC	AAG	ACA	360
106	R	R	I	Q	S	S	S	L	R	I	V	K	S	K	T	120
361	CTG	GAC	GAT	GGC	CAG	ACC	TGG	AGA	AAA	TAC	GGA	CAG	AAA	GAG	ATC	405
121	L	D	D	G	Q	T	**W**	**R**	**K**	**Y**	**G**	**Q**	**K**	E	I	135
406	CAA	AGT	GCT	AAG	CAT	CCG	AGG	AGC	TAC	TTC	AGG	TGT	ACC	CAC	AAG	450
136	Q	S	A	K	H	P	R	S	Y	F	R	**C**	T	H	K	150
451	TAC	GAT	CAA	GGT	TGC	ATG	GCG	CAC	AGA	CAG	GCG	CAG	TTA	TCA	GAG	495
151	Y	D	Q	G	**C**	M	A	H	R	Q	A	Q	L	S	E	165
496	GAC	GAT	CCC	ACT	GAT	TTC	GTG	ATC	ACA	TAC	ATC	GGG	GAG	CAC	ACC	540
166	D	D	P	T	D	F	V	I	T	Y	I	G	E	**H**	T	180
541	TGC	AGG	GAT	CCA	ACC	GTG	GTG	CCT	CCT	CAG	ATG	GTC	TCT	GCT	TCC	585
181	**C**	R	D	P	T	V	V	P	P	Q	M	V	S	A	S	195
586	ATT	TTT	CAG	GAC	ACC	TGC	CTA	ATT	AGC	TTT	GGA	GCA	GGC	GGT	CAT	630
196	I	F	Q	D	T	C	L	I	S	F	G	A	G	G	H	210
631	GGC	GTT	AGG	CAA	GAA	GCT	TCG	GTG	CCT	GCT	TCC	TTT	GCC	TCT	CAG	675
211	G	V	R	Q	E	A	S	V	P	A	S	F	A	S	Q	225
676	AAA	CAA	GAG	AGT	GAT	GAA	GAT	GCA	GCG	AGC	AAT	CTG	ACC	ACT	GCC	720
226	K	Q	E	S	D	E	D	A	A	S	N	L	T	T	A	240
721	AGT	TCG	TCG	TCG	GGC	TAT	TTC	CTG	CTA	CCA	GCG	ACC	GAG	AAT	CCG	765
241	S	S	S	S	G	Y	F	L	L	P	A	T	E	N	P	255
766	GTG	GTG	ACG	ACC	CCC	GAC	GTT	ACC	TCC	GGC	TTC	CAC	ACC	GCG	ACC	810
256	V	V	T	T	P	D	V	T	S	G	F	H	T	A	T	270
811	GAT	CTA	GAC	ATG	GAC	TTC	ATG	GCA	GAC	ACA	TAT	CTC	GAG	GAT	GTG	855
271	D	L	D	M	D	F	M	A	D	T	Y	L	E	D	V	285
856	TTC	GGG	TTC	GAT	GAC	GAC	GAG	TTC	TTC	CGA	TGA	gat	ttg	gtg	cct	900
286	F	G	F	D	D	D	E	F	F	R	*					296
901	ttc	ttg	taa	tac	tag	tgg	atc	tta	aca	gca	gag	aaa	gcc	tag	tct	945
946	gag	ggc	agc	atc	cac	cat	gtt	ccc	cag	cta	ttt	act	cag	caa	tta	990
991	att	gcc	ttc	gtt	cgc	gat	gag	gat	ctt	tcc	agc	cat	caa	cag	cac	1035
1036	agt	gac	aag	gaa	acg	ctt	gct	atc	ttc	cag	gac	aga	tca	cat	agc	1080
1081	atc	caa	gtc	aaa	tga	atg	tat	ttt	cga	gca	gca	ttt	tct	tca	tct	1125
1126	tca	acc	tcc	tgt	cga	aag	ccc	aac	ttt	ccg	aag	gct	aca	cca	tct	1170

```
1171  gta tag ttg cag aaa gat gat gac aca ctt cag agc gtg tac ttg  1215

1216  ttg gta tgc agc atc ttg atc atg tca tga aga tgc tgc tag tta  1260

1261  caa gat ctg tag aac tgt gta acc aat tta gaa ata atg atc cag  1305

1306  aaa cac ttt ttg ttc ta                                       1319
```

Figure S2. cDNA sequence of *MaWRKY45* and its deduced amino acid sequence. The WRKY domain is highlighted in gray, while the WRKYGQK motif is highlighted in blue. The amino acids of the zinc-finger motif are highlighted in red. Putative NLS residues are highlighted in orange. The 434 bp of the 3' UTR is in lower case letters. GenBank accession number: OP186310.

```
1     ATG GGG TCA AGT TGG TTG GAT TGC CCC TCT CTT AAT CTC GAC CTC   45
1      M   G   S   S   W   L   D   C   P   S   L   N   L   D   L   15

46    AAC GTC GGC TTG CTC CCG TTT CCT GTC GAT TCT CCG AAA GCT GTT   90
16     N   V   G   L   L   P   F   P   V   D   S   P   K   A   V   30

91    TCG GCC GTG GAA TCC AAG CAT GTC GAC AAG AAG GTG TCG ATC AAA   135
31     S   A   V   E   S   K   H   V   D   K   K   V   S   I   K   45

136   GAA GAG AAA GCT ATT AAG GCT CTT GGG GCG GAG CTC GTT CTG GCA   180
46     E   E   K   A   I   K   A   L   G   A   E   L   V   L   A   60

181   ACC GAA GAG AAC AAG AAG CTG AGT GAA GTG CTC GCG GCC ACG ATC   225
61     T   E   E   N   K   K   L   S   E   V   L   A   A   T   I   75

226   GCC AGC TAC AGC GCG GTT CGG AAA CAG CTG ATC GAG CAG ATG AAC   270
76     A   S   Y   S   A   V   R   K   Q   L   I   E   Q   M   N   90

271   ACC CCT CCC CCC GAA GGA GGG TCT CGA TCT AAC TCG CCG CCG GGA   315
91     T   P   P   P   E   G   G   S   R   S   N   S   P   P   G   105

316   AAG AGG AAG AGC GAG AGC CTC GAT GCT GAT ATG GAG AGC ACG TCG   360
106    K   R   K   S   E   S   L   D   A   D   M   E   S   T   S   120

361   AGC GAA GGC TCC TGC AAG CGA GTT AGA GAC GAC TGT AAA CCC AAG   405
121    S   E   G   S   C   K   R   V   R   D   D   C   K   P   K   135

406   GTC TGG AAG CTC CAT GTC CGC TCC GAC CCG TCT GAT ACA AGC CTC   450
136    V   W   K   L   H   V   R   S   D   P   S   D   T   S   L   150

451   GTC GTG AGA GAT GGG TAT CAA TGG AGG AAA TAT GGG CAG AAG GTG   495
151    V   V   R   D   G   Y   Q   W   R   K   Y   G   Q   K   V   165

496   ACC CGA GAC AAT CCA TGC CCA AGA GCT TAC TTC AGA TGC TCC TTC   540
166    T   R   D   N   P   C   P   R   A   Y   F   R   C   S   F   180

541   GCC CCT TCT TGC CCA GTT AAG AAG AAG GTA CAG AGG AGC GCG GAG   585
181    A   P   S   C   P   V   K   K   K   V   Q   R   S   A   E   195

586   GAC ACG TCG ATC TTA GTG GCG ACG TAC GAA GGC GAG CAC AAC CAT   630
196    D   T   S   I   L   V   A   T   Y   E   G   E   H   N   H   210

631   GAC CTG CGT TCT CGG CCT GGA GCT CCC AGT CTG CGT CCC AAT ACT   675
211    D   L   R   S   R   P   G   A   P   S   L   R   P   N   T   225

676   GCA GCA CCT GAC CTC AAG TCA TCA GGA TCG CAA CCG GAG ATG GAG   720
226    A   A   P   D   L   K   S   S   G   S   Q   P   E   M   E   240

721   TCG CAG GAG TTC CAG CGC AGT TTG GTG GAG CAT ATG GCT TTC TCG   765
241    S   Q   E   F   Q   R   S   L   V   E   H   M   A   F   S   255

766   TTG TCG GAA GAT CCA GCC TTC AAG GCT GCA CTG GCC ACC GCC ATC   810
256    L   S   E   D   P   A   F   K   A   A   L   A   T   A   I   270

811   TCC GGG AAA ATG CTT CCC CTG CGA ACT AGC TAA tcg ttg tcg gaa   855
271    S   G   K   M   L   P   L   R   T   S   *                   281
```

856	gat cca gcc ttc aag gct gca ctg gcc acc gcc atc tcc ggg aaa	900
901	atg ctt ccc ctg cga act agc taa ctc tct gct cct cga gat gac	945
946	tga ggt gat gcc gaa agt ttg gtt tct gtt ctt atc agt tga agt	990
991	aga cga tcc acc aat cca cag act gag ttt gat tcg gaa gaa gaa	1035
1036	gtt ccg agt tgg aca cac cga tcg atc gat ccc gaa cga aac ttc	1080
1081	ctc tcg gaa cga agg tca aaa aca cgt gct gtt gac ttc tcg gtc	1125
1126	aat gat gcc agc caa gga agt ttc gca gtt gct tat caa ggc gat	1170
1171	gtc gtt gta aat gct tta aaa gtt gtg tcg gta tga taa tgg aca	1215
1216	tcc agc cat tga ttt gtg ta	1235

Figure S3. cDNA sequence of *MaWRKY60* and its deduced amino acid sequence. The WRKY domain is highlighted in gray, while the WRKYGQK motif is highlighted in blue. The amino acids of the zinc-finger motif are highlighted in red. Putative NLS residues are highlighted in orange. The 392 bp of the 3' UTR is in lower case letters. GenBank accession number: OP186311.

1	ATG GGG AGC AGC ACA GGA AGT GAG CTC GAA AGC CCT CTC GTG GCC	45
1	M G S S T G S E L E S P L V A	15
46	GAG CTA GCT CGA GCT CTC GAG TTG GCC AGG CAG CTG GAA TCC CAT	90
16	E L A R A L E L A R Q L E S H	30
91	CTC AGC AAC CCT GCC CCA ATT GAC TTG TGC AAG TCC GTG GCG CCC	135
31	L S N P A P I D L C K S V A P	45
136	GAG ATA TTG TCA TCC ATT CAG AGG TCC ATC CTC ATG GCG AAA TCG	180
46	E I L S S I Q R S I L M A K S	60
181	AGC GAT CCC GAC GGC GAG CAG CAG GCG GCT GGC GAC AGC CCC CGC	225
61	S D P D G E Q Q A A G D S P R	75
226	AGC GAG AGT TCC AGC CCG GCG TTC AAG GAT CAC GAC CGC AAG GAG	270
76	S E S S S P A F K D H D R K E	90
271	CTG ATC AAG AAA AGG AAG ACG CTG CAC AAA TGG ACG AAT CAA GTG	315
91	L I K K R K T L H K W T N Q V	105
316	AGG CTC ACC CCG GGC ACC GGA GGA GTC GAA GGG TCT GTG GAT GAC	360
106	R L T P G T G G V E G S V D D	120
361	GGC TAC AGC TGG AGA AAG TAC GGG CAG AAG GAC ATC CTG GGA GCC	405
121	G Y S W R K Y G Q K D I L G A	135
406	AAA CAT CCG AGA GCC TAC TAC AGG TGC ACG CAC CGC CAC ACC CAG	450
136	K H P R A Y Y R C T H R H T Q	150
451	GGC TGC TCT GCG ACG AAG CAA GTG CAG AGA TCG GAC GAG GAC CCT	495
151	G C S A T K Q V Q R S D E D P	165
496	CTG ACG TTC GAC ATC ACC TAC GTC GGG GCT CAT ACC TGC CTC CAG	540
166	L T F D I T Y V G A H T C L Q	180
541	AAG CCA CAG CGA GCC TCG GCA TCC GCA TGC CAG GTG CCG CAG CGG	585
181	K P Q R A S A S A C Q V P Q R	195
586	CGG GAA CAC CAG AAG GAG GAC CTG CTG CTG AGC TTT CGA GCA GGC	630
196	R E H Q K E D L L L S F R A G	210
631	TTG AAG GTC AAG ACC GAA GTC GCG GAA TTG GAG GAA GCG CAG CGG	675
211	L K V K T E V A E L E E A Q R	225
676	CAG ACT TGT CGA CAA GGA AAT GAA TTC CAC GTC TTC TCC GCG CCG	720
226	Q T C R Q G N E F H V F S A P	240

```
721   GCC TTA AAC CTG TCG CCG GTG GCG TCG GAG TCG ATC TAC TTC AGC   765
241    A   L   N   L   S   P   V   A   S   E   S   I   Y   F   S    255

766   AGC TTT GAT GAT GGC ATC AAC CTA CAG ACT TCG GAC TCG GAG ATC   810
256    S   F   D   D   G   I   N   L   Q   T   S   D   S   E   I    270

811   ACC GAG ATG ATA TCC AGA AGC AAT TCA GCG AGC TAC TTG TCT CTG   855
271    T   E   M   I   S   R   S   N   S   A   S   Y   L   S   L    285

856   GTG GAC ATG GAT TTC ATG CTG GAG GAG CTG GAC TTC GAA CGG GAC   900
286    V   D   M   D   F   M   L   E   E   L   D   F   E   R   D    300

901   TTC CAG TTC GAT GCC TCG AGC TTC TTC TCC TGA gtc atg gga cat   945
301    F   Q   F   D   A   S   S   F   F   S   *                    311

946   gag cag agt cca aac act cga gat cgc tca att cat ttt ccg aga   990

991   tta aac aag acg act ggt aaa ttc ccc aac cgg aaa tgt agg tag   1035

1036  aaa cag aaa gag ttt gtt cca tgt aat gcg tag ctc aat ttt gtg   1080

1081  tta aaa tca atg gat caa gtc a                                 1102
```

Figure S4. cDNA sequence of *MaWRKY70* and its deduced amino acid sequence. The WRKY domain is highlighted in gray, while the WRKYGQK motif is highlighted in blue. The amino acids of the zinc-finger motif are highlighted in red. Putative NLS residues are highlighted in orange. The 169 bp of the 3’ UTR is in lower case letters. GenBank accession number: OP186312.

Supplementary tables

Table S1. List of primers for RT-PCR and RT-qPCR.

**Gene**	**Forward primer (5'→3')**	**Reverse primer (5'→3')**	**Amplicon size**	**Reference**
		Coding sequence amplification by RT-PCR		
MaWRKY18	ATGGGATCGGCTTGGTTG	TTAAAGCATCATCCCGGAAATG	828	This study
MaWRKY45	ATGGAACACACGCCTGTTG	TCATCGGAAGAACTCGTCG	888	This study
MaWRKY60	ATGGGGTCAAGTTGGTTGG	TTAGCTAGTTCGCAGGGGAAG	843	This study
MaWRKY70	ATGGGGAGCAGCACAGG	TCAGGAGAAGAAGCTCGAGG	933	This study
		RT-qPCR		
MaWRKY18	AGGTCTTCGCATCCCAAGTC	AAAGCGTCACAGAGAAGAAGCC	192	This study
MaWRKY45	TTCATGGCAGACACATATCTCG	TGCTGAGTAAATAGCTGGGGAAC	161	This study
MaWRKY60	TCGTTGTCGGAAGATCCAGC	TCCGAATCAAACTCAGTCTGTG	185	This study
MaWRKY70	ATGGATTTCATGCTGGAGGAG	TGTTTCTACCTACATCACCAGTTGG	176	This study
MaDLO1	GAGAGCTTGGGACTTGAGAAG	CTGTGGGCATGGTG GATAG	93	Tzean *et al*.,2021
MaLOX1	CCCAACAGCGTGTCCATCTA	CTTTGCGTCCAAGCCACCAC	202	Zhao *et al*., 2013
Ma25S	ACATTGTCAGGTGGGGAGTT	CCTTTTGTTCCACACGAGATT	106	Tzean *et al*.,2021

Table S2. List of forward primers for 3′ UTR amplification using RACE.

**Gene**	**First-round PCR**	**Second-round PCR**	**Reference**
MaWRKY18	ACAGCCAGCCTTCTCAGGTC	AGGTCTTCGCATCCCAAGTC	This study
MaWRKY45	AAGATGCAGCGAGCAATCTGACC	TTCCTGCTACCAGCGACCGAG	This study
MaWRKY60	ACCGGAGATGGAGTCG	TCGTTGTCGGAAGATCCAGC	This study
MaWRKY70	TGGCGTCGGAGTCGATCTACTTC	ACAGACTTCGGACTCGGAGATCACC	This study

Table S3. Best BLASTP hits of rice OsWRKY45 and *Arabidopsis* AtWRKY70 protein sequences used as queries in the proteome of *M. acuminata* ssp. *malaccensis*.

Query	Genbank accession number	Best BlastP hits in banana proteome	Length of BlastP alingment	Identity %	Similarity %	Bitscore	Expected (*E*) value
OsWRKY45	AK066255	GSMUA_Achr5P07490	177	44	55	123.25	1.78e-032
AtWRKY70	AF421157	GSMUA_Achr1P27980	73	55	74	102.449	2.98e-25

Table S4. Best BLASTP hits of banana GSMUA_Achr3P13440 and GSMUA_Achr7P05200 protein sequences used as queries in the *Arabidopsis* proteome.

Query	Best BlastP hits in *Arabidopsis* proteome	Length of BlastP alingment	Identity %	Similarity %	Bitscore	Expected (*E*) value
GSMUA_Achr3P13440	AtWRKY18	306	41	52	183.726	1.81e-56
GSMUA_Achr7P05200	AtWRKY60	263	49	60	187.963	1.76e-58

Table S5. Amino acid sequence similarity (%) between banana MaWRKY18, MaWRKY45, MaWRKY60, MaWRKY70 and their respective homologs in *Arabidopsis* and rice.

**WRKY**	MaWRKY18	MaWRKY45	MaWRKY60	MaWRKY70	AtWRKY18	OsWRKY45	AtWRKY60	AtWRKY70
MaWRKY18		27.5	67.9	23.0	50.6	15.2	29.8	15.7
MaWRKY45	…		19.5	21.0	17.5	25.5	18.2	20.3
MaWRKY60	…	…		16.0	35.3	14.7	28.8	14.0
MaWRKY70	…	…	…		16.0	24.8	14.2	22.3
AtWRKY18	…	…	…	…		14.5	46.8	16.0
OsWRKY45	…	…	…	…	…		13.5	22.8
AtWRKY60	…	…	…	…	…	…		14.7
AtWRKY70	…	…	…	…	…	…	…	

% similarity between banana MaWRKY18, MaWRKY45, MaWRKY60, MaWRKY70 and their putative orthologs are highlighted in gray.
